# Multidimensional Vibration Suppression Method with Piezoelectric Control for Wind Tunnel Models †

**DOI:** 10.3390/s19183998

**Published:** 2019-09-16

**Authors:** Mengde Zhou, Wei Liu, Linlin Tang, Zhuang Yao, Zhengquan Wen, Bing Liang, Zhenyuan Jia

**Affiliations:** Key Laboratory for Precision and Non-traditional Machining Technology of the Ministry of Education, Dalian University of Technology, Dalian 116024, China; mengde@mail.dlut.edu.cn (M.Z.); Tanglinlin1995@mail.dlut.edu.cn (L.T.); yaozhuang@mail.dlut.edu.cn (Z.Y.); wenzhengquan@mail.dlut.edu.cn (Z.W.); liangbing2016@mail.dlut.edu.cn (B.L.); zhenyuanjia@yeah.net (Z.J.)

**Keywords:** wind tunnel, multidimensional active control, damping, stackable piezoelectric actuator, accelerometer

## Abstract

In wind tunnel tests, the low-frequency and large-amplitude vibration of the cantilever sting result in poor test data in pitch plane and yaw plane, more seriously, even threatens the safety of wind tunnel tests. To ensure the test data quality, a multidimensional vibration suppression method is proposed to withstand the vibration from any direction, which is based on a system with stackable piezoelectric actuators and velocity feedback employing accelerometers. Firstly, the motion equation of the cantilever sting system is obtained by Hamilton’s principle with the assumed mode method. Then, the multidimensional active control mechanism is qualitatively analyzed and a negative velocity feedback control algorithm combined with a root mean square (RMS) evaluation method is introduced to realize active mass and active damping effect, meanwhile, a weight modification method is performed to determine the sequence number of the stacked piezoelectric actuators and the weight of control voltages in real time. Finally, a multidimensional vibration suppression system was established and verification experiments were carried out in lab and a transonic wind tunnel. The results of lab experiments indicate that the damping ratio of the system is improved more than 4.3 times and the spectrum analyses show reductions of more than 23 dB. In addition, wind tunnel test results have shown that for the working conditions (α = −4~10° with γ = 0° or α = −4~10° with γ = 45°) respectively at 0.6 Ma and 0.7 Ma, the remainder vibration is less than 1.53 g, which proves that the multidimensional vibration suppression method has the ability to resist vibration from any direction to ensure the smooth process of wind tunnel tests.

## 1. Introduction

Wind tunnel test plays an important role in developing the aircraft. To reduce the aerodynamic interference of sting support system on test section flow around aircraft model, the length of the cantilever sting, especially in transonic tunnels, usually varies from about three to five times the length of the aircraft model [1]. It is a long cantilever beam with low structural damping. As aircraft model sweeps to big angle-of-attack, flow separation appears over the wings and buffeting [2] causes a kind of low-frequency and big-amplitude vibration at the first resonant frequency [3], which results in poor balance data quality in pitch plane and yaw plane [4]. As a result, the balance works disorderly and the test safety will suffer the serious threat if the test is not stopped immediately [5,6]. Therefore, the vibration control methods and systems to ensure the tests data quality and the safety of wind tunnel tests have paid more attention to such wind tunnel tests condition.

Some research works have been carried out on the vibration control problems of aircraft model in wind tunnel test. So far, two main methods have been reported. They are the passive vibration control (PVC) method and the active vibration control (AVC) method [7]. Igoe et al. [8] first proposed the PVC method using a passive device installed inside the aircraft model without power input to reduce the support system vibration. However, the effectiveness in yaw plane was restricted. In pitch plane, the vibration was only reduced by approximately 50% at some wind-tunnel test conditions. With the advent of piezoelectric materials, the AVC method using piezoelectric materials as actuator has been rapidly and widely applied in aeronautics field [9,10,11].

Fehren [1,12,13] first proposed an anti-vibration system (AVS) to counterbalance the vibration of aircraft model. It is the first time to realize active vibration control for aircraft model in wind tunnel using integrated piezoelectric elements controlled by a digital control system with the balance signals. However, due to its structural limitations, only about 60% of vibration control effect was achieved and the AVS system was less effective for strong vibrations in pitch plane. To further research, Balakrishna et al. [1,14] developed a damper based on the AVS in ViGYAN’s low-speed wind tunnel which depended on balance signals and test measurement system in wind tunnel. The damping coefficient enhanced three times and spectral analysis of the response shows an attenuation of 10 dB with active control in yaw plane. Many other research works have also been conducted mainly focusing on the application of the system. Acheson et al. [15] applied the vibration control technology to common research model (CRM) at the NASA Langley Research Center National Transonic Facility (NTF), and on the aspects of damper design, the CRM model was detailed from a sting damping energy viewpoint. Balakrishna et al. [16] and Rivers et al. [17] studied the influence of the piezoelectric damper location on the vibration control effect at the NASA Langley Research Center National Transonic Facility (NTF) and Ames Research Center 11 × 11 Foot, the model vibration can be reduced with an average vibration attenuation of about 5 dB and peak attenuation of about 10 dB. However, although the proposed systems initially achieved the vibration control of aircraft model, the control effect in pitch and yaw plane is limited. Therefore, it is necessary to theoretically analyze the model of active vibration control system with stackable piezoelectric actuators, and then study the active vibration control algorithm. Xie et al. [11] proposed a gust load alleviation (GLA) control technique employing piezoelectric patches in the research on alleviating the transverse gust-response loads of a large-aspect-ratio flexible wing on the basis of modeling with Hamilton’s principle. The gust load alleviation rate is generally over 20%. Song et al. [18] applied piezoelectric patch as actuator/sensor to study the active aeroelastic flutter characteristics and vibration control of supersonic beams, based on dynamic analysis with Hamilton’s principle, the active aeroelastic flutter suppression is achieved for the supersonic beams, especially at the flutter points. Shen et al. [19,20,21] aiming at the vibration of wind tunnel aircraft model in pitch plane, introduced an active damping system and control strategy using stacked piezoelectric actuators and balance signal. Three algorithms, respectively, classical PD algorithm, artificial neural network PID (NNPID) and linear quadratic regulator (LQR) optimal control algorithm were discussed. The results show that the algorithms can achieve the purpose of over 80% reduction.

As mentioned above, most studies focus on the active control method and algorithm of transverse vibration using piezoelectric patch as actuator and wind tunnel balance signal as feedback. In this paper, focusing on the vibration from any direction affecting the test data quality in pitch plane and yaw plane, on the basis of system modeling and dynamic analysis, a multidimensional vibration suppression method and system are proposed employing stacked piezoelectric actuators and velocity feedback with accelerometers. 

The remainder of this paper is organized as follows. Section 2 introduces the multidimensional vibration suppression system and method against the vibration from any direction employing stackable piezoelectric actuators and velocity feedback using accelerometers. Section 3 presents the motion equation of the cantilever sting system obtained by Hamilton’s principle with the assumed mode method. In Section 4, the multidimensional active control mechanism is qualitatively analyzed, meanwhile, a negative velocity feedback control algorithm combined with a root mean square (RMS) evaluation method and a weight modification method are introduced to strengthen the damping and mass characteristics of the cantilever sting system. In Section 5, a multidimensional vibration suppression system was established and verification experiments were performed. Section 6 summarizes this paper.

## 2. Multidimensional Vibration Suppression System and Method

**Figure 1:**
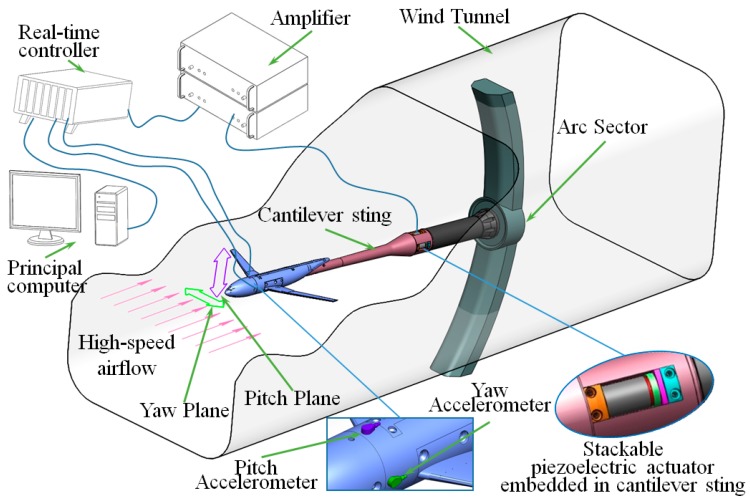
Schematic of multidimensional vibration suppression principle for wind tunnel models.

The multidimensional vibration suppression method is shown in Figure 1 focusing on the vibration in any direction affecting the test data quality in pitch plane and yaw plane, a multidimensional vibration suppression method and system are proposed employing stacked piezoelectric actuators and velocity feedback with accelerometers.

With this method, two accelerometers are installed on the barycenter of aircraft model in pitch plane and yaw plane to increase the universality of the system and avoid ineffectual obtaining of workable vibration data from wind tunnel balance, especially under intense vibration or in the wind tunnel tests without wind tunnel balance. Then the real-time velocities of the vibrating aircraft model can be obtained by integrating the acceleration signal. The vibration velocities in pitch and yaw planes are fed back to the real-time controller in real time. Then, on the basis of system modeling and dynamic analysis, a closed-loop controller with velocities as feedback is developed on the development platform of real-time controller. Finally, the four stacked piezoelectric actuators are controlled through two two-channel amplifiers to withstand the stochastic dynamic wind load in real time.

## 3. System Modeling

In this section, the stochastic dynamic wind load with broadband is simplified in pitch plane and yaw plane, in which the data quality is seriously affected. The motion equation of the cantilever sting embedded with stackable piezoelectric actuators is established by Hamilton’s principle with the assumed mode method, which lays a foundation for the study of multidimensional vibration suppression method.

**Figure 2:**
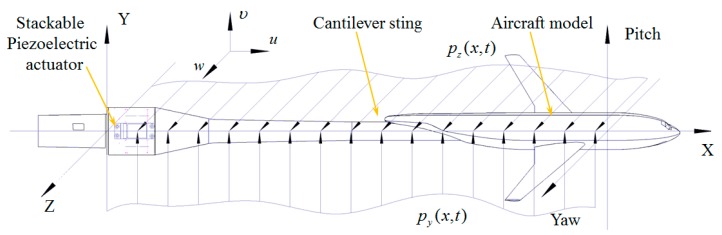
Schematic diagram of cantilever sting with stackable piezoelectric actuators.

Figure 2 shows the cantilever sting with stackable piezoelectric actuators in the Cartesian coordinates. The displacements in pitch plane and yaw plane are considered in this study. The Euler–Bernoulli beam theory is used in this paper. The relationship between stress and strain is written as

(1) $$\sigma_{x}=E\varepsilon_{x},\;\varepsilon_{x}=-y\frac{\partial^{2}\upsilon }{\partial x^{2}}-z\frac{\partial^{2}w}{\partial x^{2}}$$

where $\sigma_{x}$ and $\varepsilon_{x}$ are the stress and strain in the x direction. υ and w are the displacements of the cantilever sting in pitch plane and yaw plane, and E is the modulus of elasticity of the cantilever sting.

For the stackable piezoelectric actuator, the polarization direction of each piezoelectric patch is in the x axis. Applying the same external voltage to each piezoelectric patch, the constitutive equations are written as [22]

(2) $$\{\begin{array}{c} \varepsilon_{x}=nC^{E}\sigma_{x}^{p}+ndE_{x}\\ D_{x}=nd\sigma_{x}^{p}+n\varepsilon^{\sigma }E_{x} \end{array}$$

where $\sigma_{x}^{p}$ is the stress, $C^{E}$ is the elastic compliance constant, d is the piezoelectric strain constant, $E_{x}=u_{0}/th$ is the electric intensity along the x direction, $D_{x}$ is the electric displacement, $\varepsilon^{\sigma }$ is the dielectric constant, $u_{0}$ is the external applied voltage, th is the thickness of each piezoelectric patch, and n is the number of piezoelectric patches in the stackable piezoelectric actuator.

Hamilton’s principle with the assumed mode method is used to determine the motion equation of the cantilever sting with stackable piezoelectric actuators system, the principle is written as

(3) $$\delta \int_{t_{1}}^{t_{2}}(T-U)\;dt+\int_{t_{1}}^{t_{2}}\delta Wdt=0$$

where T and U are the total kinetic energy and potential energy of the cantilever sting with stackable piezoelectric actuators, δW is the virtual work done by external loads, δ(⋅) indicates the first variation, $t_{1}$ and $t_{2}$ are the integration time limits. 

The total kinetic energy of the whole system consists of four parts which are the kinetic energy of the cantilever sting and stacked piezoelectric actuators respectively in pitch plane and yaw plane, the total kinetic energy can be expressed as

(4) $$T=\frac{1}{2}\underset{V_{b}}{∭}\rho_{b}\left[{\left(\frac{\partial \upsilon }{\partial t}\right)}^{2}+{\left(\frac{\partial w}{\partial t}\right)}^{2}\right]\;dV+\frac{1}{2}\underset{V_{p}}{∭}\rho_{p}\left[{\left(\frac{\partial \upsilon }{\partial t}\right)}^{2}+{\left(\frac{\partial w}{\partial t}\right)}^{2}\right]\;dV$$

where $\rho_{b}$ and $\rho_{p}$ are the densities of the cantilever sting and stackable piezoelectric actuator, respectively. $V_{b}$ and $V_{p}$ are the volumes of the cantilever sting and stackable piezoelectric actuator, respectively.

The total potential energy of the whole system consists of three parts. They are strain energies of the cantilever sting and stacked piezoelectric actuators and electric potential energy of the stacked piezoelectric actuators, then the total potential energy can be expressed as

(5) $$U=\frac{1}{2}\underset{V_{b}}{∭}\sigma_{x}\varepsilon_{x}dV+\frac{1}{2}\underset{V_{p}}{∭}\sigma_{x}^{p}\varepsilon_{x}dV-\frac{1}{2}\underset{V_{p}}{∭}D_{x}E_{x}dV$$

The external work is caused by the unsteady aerodynamics of the random wind load acting on the aircraft model in the pitch and yaw plane, the virtual work can be expressed as

(6) $$\delta W=\int_{0}^{l}\left[p_{y}(x,t)\delta \upsilon +p_{z}(x,t)\delta w\right]\;dx$$

where $p_{y}(x,t)$ and $p_{z}(x,t)$ are the distributed loads acting on the aircraft model and the cantilever sting, respectively, in pitch plane and yaw plane.

With the assumed mode method, in terms of generalized coordinates, the pitch displacement and yaw displacement can be expressed as

(7) $$\upsilon (x,t)=\sum_{i=1}^{n}\nu_{i}(x)q_{yi}(t)=\nu^{T}(x)q_{y}(t)$$

(8) $$w(x,t)=\sum_{i=1}^{n}w_{i}(x)q_{zi}(t)=w^{T}(x)q_{z}(t)$$

where $q_{y}(t)={\left[\begin{array}{ccc} q_{y1} & \cdots & q_{yn} \end{array}\right]}^{T}$ and $q_{z}(t)={\left[\begin{array}{ccc} q_{z1} & \cdots & q_{zn} \end{array}\right]}^{T}$ are the generalized coordinates of the cantilever sting with stacked piezoelectric actuators, and $\nu (x)={\left[\begin{array}{ccc} \nu_{1} & \cdots & \nu_{n} \end{array}\right]}^{T}$ and $w(x)={\left[\begin{array}{ccc} w_{1} & \cdots & w_{n} \end{array}\right]}^{T}$ are the principal vibration mode shapes satisfied the geometric boundary conditions.

Substituting (1)–(2) and (7)–(8) for (4)–(6), the kinetic energy, potential energy and virtual work are expressed in terms of the normal modes and the generalized coordinate as

(9) $$T=\frac{1}{2}{\dot{q}}_{y}^{T}M_{y}{\dot{q}}_{y}+\frac{1}{2}{\dot{q}}_{z}^{T}M_{z}{\dot{q}}_{z}$$

(10) $$U=\frac{1}{2}q_{y}^{T}K_{y}q_{y}^{T}+\frac{1}{2}q_{z}^{T}K_{z}q_{z}^{T}+V_{0y}K_{s\phi }q_{y}+V_{0z}K_{s\phi }q_{z}+\frac{1}{2}K_{0y}V_{0y}^{2}+\frac{1}{2}K_{0z}V_{0z}^{2}$$

(11) $$\delta W=p_{y}\delta q_{y}+p_{z}\delta q_{z}$$

where $M_{y}$, $M_{z}$, $K_{y}$, $K_{z}$ are the modal masses and stiffness matrices of the cantilever sting and stackable piezoelectric actuator respectively in pitch plane and yaw plane, $K_{y\phi }$, $K_{z\phi }$, $K_{0y}$, $K_{0z}$ are the electromechanical coupling matrices and the piezoelectric capacitances of the stackable piezoelectric actuator respectively in pitch plane and yaw plane, and $p_{y}$ and $p_{z}$ are the forcing matrices in pitch plane and yaw plane, respectively.

Substituting (9)–(11) for (3), performing the variation operation in terms of $q_{y}^{}(x)$ and $q_{z}^{}(x)$, the dynamic motion equation of the cantilever sting with stackable piezoelectric actuator is obtained. That is

(12) $$M\ddot{X}(t)+C\dot{X}(t)+KX(t)=F+Bu_{0}(t)$$

where M, C, K, F and B are the modal mass matrix, damping matrix, stiffness matrix, generalized stochastic aerodynamic loads and generalized driving matrix of stackable piezoelectric actuators. These matrices are expressed as

(13) $$X(t)=\left[\begin{array}{c} q_{y}^{}(x)\\ q_{z}^{}(x) \end{array}\right],\;M=\left[\begin{array}{cc} M_{y} & 0\\ 0 & M_{z} \end{array}\right],\;K=\left[\begin{array}{cc} K_{y} & 0\\ 0 & K_{z} \end{array}\right],\;C=\left[\begin{array}{cc} C_{y} & 0\\ 0 & C_{z} \end{array}\right]$$

Equation (12) is called the actuator equation, which characterizes the stackable piezoelectric actuators driven under the externally applied voltage $u_{0}$ and it relates the applied voltage to the structural displacements. Equation (12) is used to study vibration suppression by a negative velocity feedback control method.

## 4. Multidimensional Active Vibration Control

In this section, on the basis of structural modeling of cantilever sting with stackable piezoelectric actuators, the multidimensional vibration suppression method is analyzed and a negative velocity feedback control algorithm combined with a root mean square (RMS) evaluation method is introduced to realize active mass and active damping effect, meanwhile, a weight modification method based on the stress $\sigma_{x}^{p}$ is performed to determine the sequence number of the stacked piezoelectric actuators and the weight of control voltages in real time.

### 4.1. Active Control Architecture

**Figure 3:**
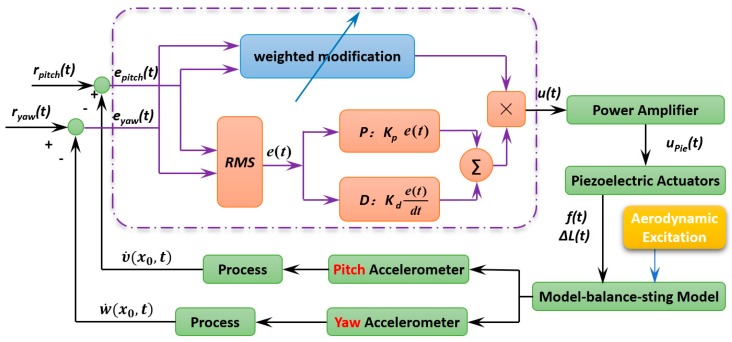
Schematic of control system.

As shown in Figure 3, the multidimensional vibration suppression method is based on feedback loop and a negative velocity feedback control algorithm is used. Velocity $\dot{\upsilon }(x_{0},t)$ and $\dot{w}(x_{0},t)$ at the position $x_{0}$ are the measured output of active vibration closed loop control system, $r_{pitch}(t)$, $r_{yaw}(t)$, $e_{pitch}(t)$, and $e_{yaw}(t)$ are defined as the value of vibration target, the output error in pitch plane and yaw plane, respectively. Then, the output error respectively in pitch plane and yaw plane are defined as

(14) $$\{\begin{array}{c} e_{pitch}(t)=r_{pitch}(t)-\dot{\upsilon }(x_{0},t)\\ e_{yaw}(t)=r_{yaw}(t)-\dot{w}(x_{0},t) \end{array}$$

Vibration from any direction makes the stresses on each stacked piezoelectric actuators not only vary from each other, but also change in real time. In this paper, according to the sign of stresses on each stacked piezoelectric actuators, the sequence number of the stacked piezoelectric actuators participating in vibration control is determined, and the weight of control voltages is determined by the force. According to the theory of material mechanics, the bending moment acting on the section of stacked piezoelectric actuators in pitch plan and yaw plane can be respectively expressed as

(15) $$M_{pitch}(t)=-m_{eq}\cdot \frac{e_{pitch}(t)}{d(t)}\cdot L,M_{yaw}(t)=-m_{eq}\cdot \frac{e_{yaw}(t)}{d(t)}\cdot L$$

where $m_{eq}$ is the equivalent mass, L is the distance from the barycenter of aircraft model to the end faces of stacked piezoelectric actuators.

Then, stresses acting on the end faces of stacked piezoelectric actuators can be calculated as

(16) $$\sigma_{xi}^{p}=\frac{M_{pitch}(t)\cdot d}{I_{p}(t)},\;i=1,3,\;\sigma_{xi}^{p}=\frac{M_{yaw}(t)\cdot d}{I_{p}(t)},\;i=0,2$$

where d is the distance from stacked piezoelectric actuators to the neutral layer, $I_{p}(t)$ is the inertial moment of cantilever sting in the end face of stacked piezoelectric actuators.

Thus, the force acting on the stackable piezoelectric actuators can be expressed as

(17) $$F_{NRi}\left(t\right)=\iint_{A_{i}}\sigma_{xi}^{p}dA_{i},i=0,1,2,3$$

where i is the sequence number of the stacked piezoelectric actuators. If $F_{NRi}\left(t\right)>0$, the stacked piezoelectric actuator participates in active vibration control. Further, the weight of control voltage is determined by the force, the weight matrix of control voltages is written as

(18) $$R\left(t\right)=\frac{1}{K_{pie}}F_{NR}\left(t\right)$$

where $K_{pie}$ is the load rigidity coefficient, and $F_{NR}\left(t\right)={\left[\begin{array}{ccc} F_{NR0}\left(t\right) & \cdots & F_{NRi}\left(t\right) \end{array}\right]}^{T},i=0,1,2,3$ is the output force matrix.

To strengthen the damping and mass characteristics of the cantilever sting, a velocity feedback controller with proportional and derivative is applied on the stackable piezoelectric actuators, the modulus of system input voltage is expressed as

(19) $$K(t)=K_{p}e(t)+K_{d}\frac{de(t)}{dt}$$

where $K_{p}$ and $K_{d}$ are the proportional gain and derivative gain, respectively.

In order to better evaluate the vibration contributions to principal vibration from pitch plane and yaw plane, the root mean square (RMS) method is introduced, and output error can be written as

(20) $$e(t)=\sqrt{(e_{pitch}^{2}(t)+e_{yaw}^{2}(t))/2}$$

Finally, the system input voltage matrix is expressed as

(21) $$u(t)=K_{A}K(t)R(t)$$

where $K_{A}$ is the amplification coefficient of power amplifier.

Equation (21) is the externally applied voltage equation applying to the stackable piezoelectric actuators by a negative velocity feedback control method.

### 4.2. Active Control Analysis

Two of the four stackable piezoelectric actuators participate in vibration control in each half of the vibration cycle. The external voltages in every half cycle can be written as

(22) $$\{\begin{array}{c} u_{0y}(t)=-K_{py}\dot{\upsilon }(x_{0},t)-K_{dy}\ddot{\upsilon }(x_{0},t)\\ u_{0z}(t)=-K_{pz}\dot{w}(x_{0},t)-K_{dz}\ddot{w}(x_{0},t) \end{array}$$

where $K_{py}=K_{A}K_{p}R_{pitch}\left(t\right)$ and $K_{dy}=K_{A}K_{d}R_{pitch}\left(t\right)$ are the feedback control gains in pitch plane, $K_{pz}=K_{A}K_{p}R_{yaw}\left(t\right)$ and $K_{dz}=K_{A}K_{d}R_{yaw}\left(t\right)$ are the feedback control gains in yaw plane, and $\upsilon (x_{0},t)$ and $w(x_{0},t)$ are the displacements respectively in pitch plane and yaw plane at position $x_{0}$ on the aircraft model.

Substituting (7)–(8) for (22), the external voltages can be expressed as

(23) $$u_{0}(t)=-K_{p}\left[\begin{array}{cc} \nu^{T}(x_{0}) & 0\\ 0 & w^{T}(x_{0}) \end{array}\right]\left[\begin{array}{c} {\dot{q}}_{y}\\ {\dot{q}}_{z} \end{array}\right]-K_{d}\left[\begin{array}{cc} \nu^{T}(x_{0}) & 0\\ 0 & w^{T}(x_{0}) \end{array}\right]\left[\begin{array}{c} {\ddot{q}}_{y}\\ {\ddot{q}}_{z} \end{array}\right]=-K_{p}\Phi \dot{X}-K_{d}\Phi \ddot{X}$$

The coefficient matrix can be written as

(24) $$\Phi =\left[\begin{array}{cc} \nu^{T}(x_{0}) & 0\\ 0 & w^{T}(x_{0}) \end{array}\right]$$

Substituting (23) for (12), the motion equation with active damping and active mass is obtained as

(25) $$(M+BK_{d}\Phi )\ddot{X}(t)+(C+BK_{p}\Phi )\dot{X}(t)+KX(t)=F$$

The active mass matrix and active damping matrix related to stacked piezoelectric actuators can be written as

(26) $$M_{A}=M+BK_{d}\Phi ,\;C_{A}=C+BK_{p}\Phi$$

It is seen from Equation (25) that the velocity feedback control algorithm provides the active mass and active damping to the cantilever sting.

## 5. Verification Experiments in Lab and Wind Tunnel

In this section, the proposed multidimensional vibration suppression method and system for wind tunnel models is verified by experiments in lab and wind tunnel.

### 5.1. Experimental System

**Figure 4:**
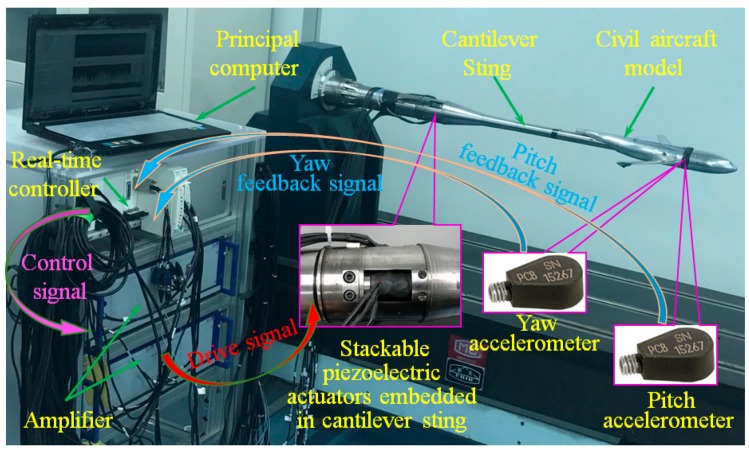
Diagram of multidimensional vibration suppression system.

As shown in Figure 4, a civil aircraft model was used to verify the multidimensional vibration suppression system. The verification system consists of two Integrated Electronics Piezo Electric (IEPE) accelerometers, a NI real-time controller, two two-channel amplifiers, a cantilever sting with pre-tightening configurations and four stackable piezoelectric actuators. Among those, the NI real-time controller includes acceleration acquisition board (NI PXI-4461 with 12 bit resolution), I/O boards (NI PXI-6363/5922), and central processing unit (PXIe-1071DC), the four stackable piezoelectric actuators were embedded in the cantilever sting, and the pre-tightening mechanisms were employed to guarantee the reliable output of dynamic force. Besides, parameters of stackable piezoelectric actuator are shown in Table 1.

### 5.2. Impulse Verification Experiments in Lab

**Figure 5:**
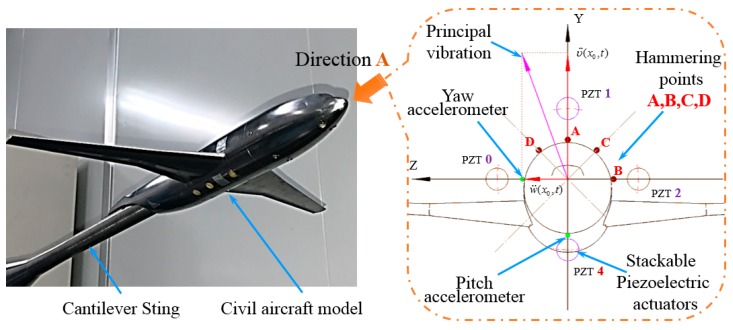
Distribution diagram of the hammering points (Direction A).

**Figure 6:**
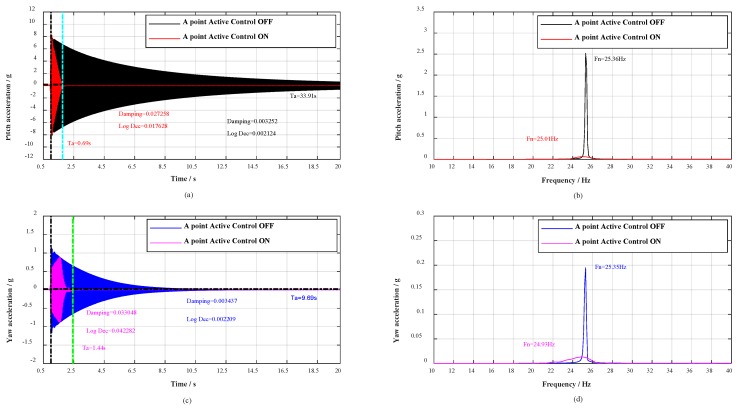
Response spectral analysis. Control effect during impulse tests in pitch plane (A point).

On the basis of the multidimensional vibration suppression system, verification experiments were conducted. In the experiment, the broadband stochastic dynamic wind loads were simulated through hammering. The distribution diagram of the hammering points A, B, C, D is shown from A direction in Figure 5. As flow separation mainly occurs on the wing surface, buffeting causes the low-frequency and big-amplitude vibration in pitch plane. Therefore, a series of hammerings at A point were carried out to verify the vibration control effect in pitch plane, the contrast effects with multidimensional vibration suppression system off and on are shown in Figure 6. 

**Figure 7:**
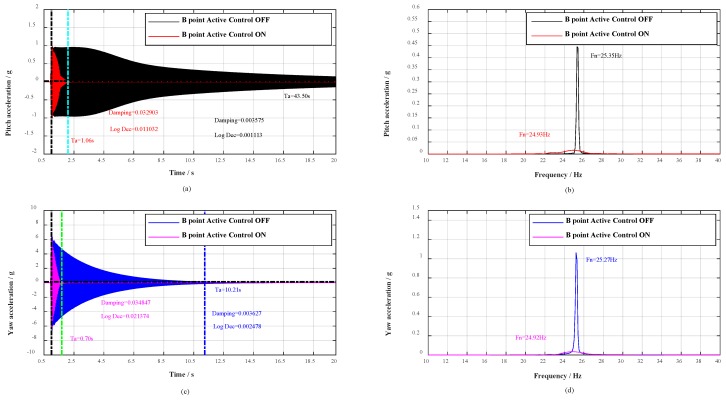
Response spectral analysis. Control effect during impulse tests in yaw plane (B point).

**Figure 8:**
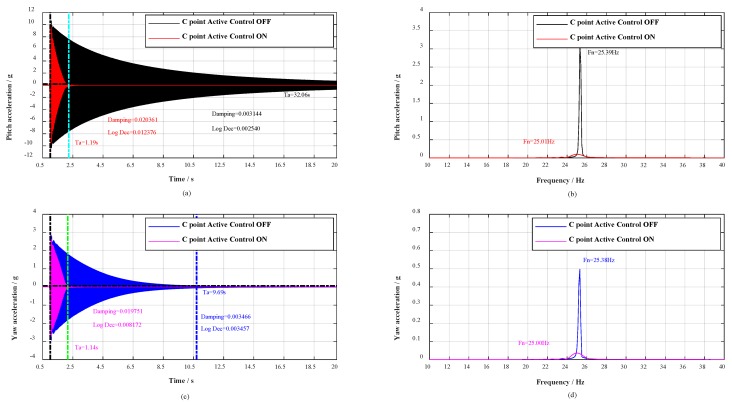
Response spectral analysis. Control effect during impulse tests in any plane (C point).

**Figure 9:**
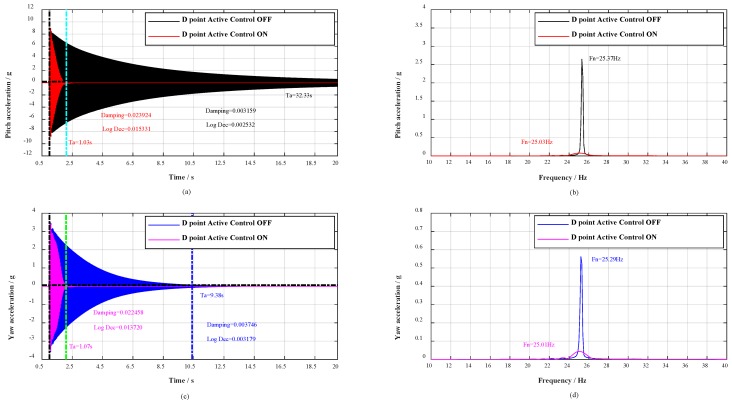
Response spectral analysis. Control effect during impulse tests in any plane (D point).

Moreover, with the change of roll angle γ during the wind tunnel tests, the aircraft model vibrates in any plane, concurrently, the adjoint vibration appears in yaw plane. Thus, a great deal of hammerings at B point were first carried out to verify the vibration control effect in yaw plane, and then respectively hammering at C point and D point to verify the vibration control effect in any plane. The contrast effects with multidimensional vibration suppression system off and on are shown in Figure 7, Figure 8 and Figure 9. 

As shown in Figure 6a,c, when the civil aircraft model was hammered at A point, the vibrations were damped within 0.69 and 1.44 s respectively in pitch plane and yaw plane. Whereas, the vibrations in pitch plane and yaw plane respectively last for over 33.91 s and 9.69 s without multidimensional vibration suppression system. At the same time, the damping ratio in pitch plane calculated by half-power is strengthened 8.38 times (from 0.003252 to 0.027258) and 9.62 times (from 0.003437 to 0.033048) in yaw plane. Evaluating from logarithmic decrement method, the damping ratio in pitch plane is strengthened 8.30 times (from 0.002124 to 0.016728) and 10.99 times (from 0.002209 to 0.024282) in yaw plane. The spectrum analysis for vibrations is shown in Figure 6b,d, the spectrum analysis for the impulse response in pitch plane shows a reduction of 32 dB and the natural frequency decreased from 25.36 to 25.01 Hz, as well as in yaw plane, the spectral analysis shows a reduction of 23 dB and the natural frequency decreased from 25.35 to 24.94 Hz. Besides, when the civil aircraft model was hammered at B, C, and D, the verification results are listed in Table 2.

For any point (A, B, C, or D), the vibrations are damped within 1.44 s, the damping ratio is strengthened more than 4.3 times, and the spectral analysis of vibrations shows attenuations of more than 23 dB. According to the analysis above, the damping and mass characteristics of the cantilever sting are strengthened. 

### 5.3. Verification Experiments in Wind Tunnel

**Figure 10:**
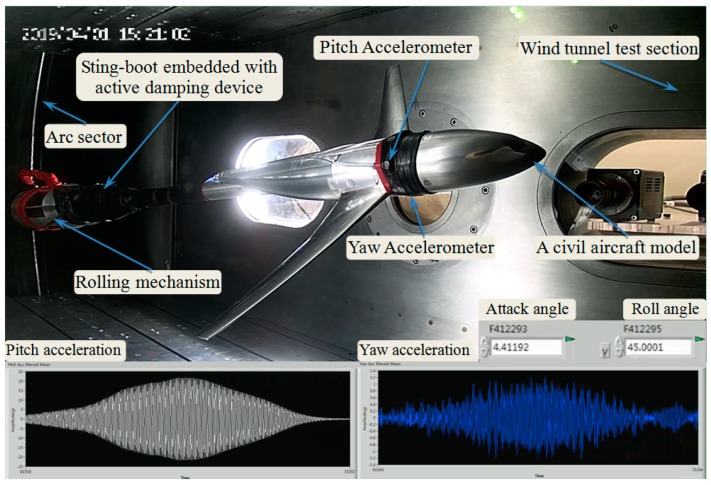
Photograph of the multidimensional vibration suppression system installed in a continuous transonic wind tunnel.

The wind tunnel verification experiments were carried out in a continuous transonic wind tunnel. As shown in Figure 10, a civil aircraft model was mounted on the sting embedded with active damping device through a wind tunnel balance, and the model-balance-sting system was installed on the attack-roll-angle adjusting mechanism consisting of arc sector and rolling mechanism. Two accelerometers were set on the pitch plane and yaw plane of the civil aircraft model, and the pitch acceleration, the yaw acceleration, attack angle, and roll angle were monitored and stored by the real-time controller. 

**Figure 11:**
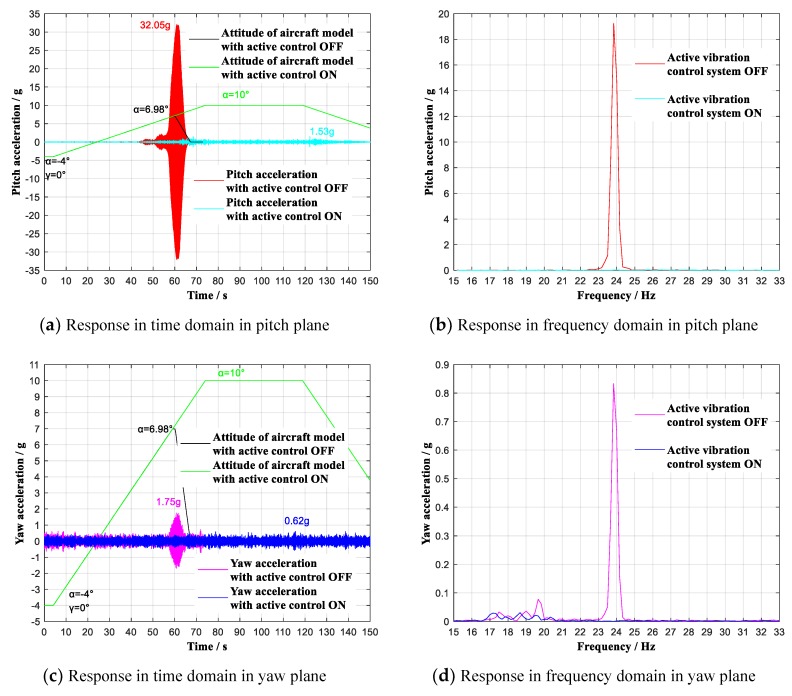
Vibration with multidimensional vibration suppression system on and off lifting continuous (γ = 0°) at 0.6 Ma. (**a**) Response in time domain in pitch plane; (**b**) Response in frequency domain in pitch plane; (**c**) Response in time domain in yaw plane; (**d**) Response in frequency domain in yaw plane.

**Figure 12:**
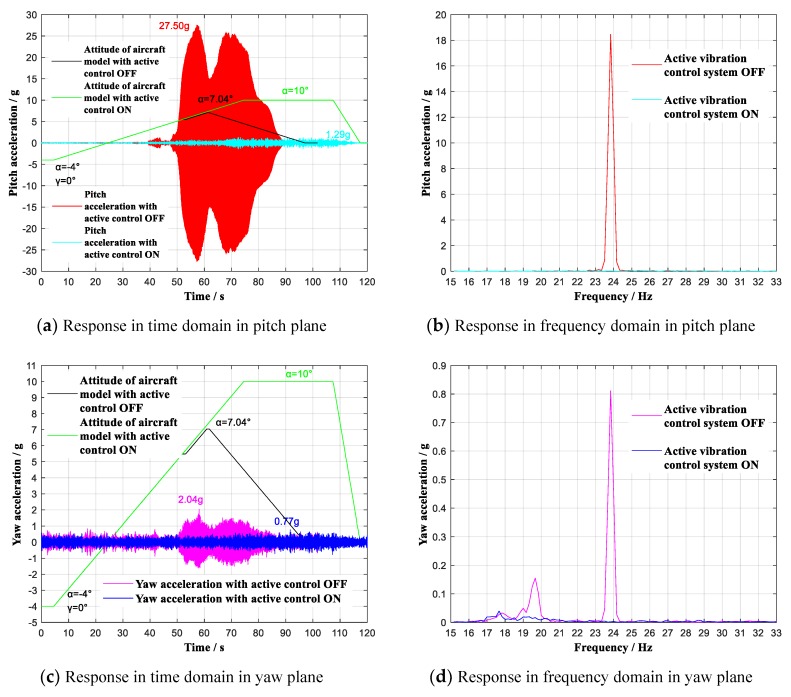
Vibration with multidimensional vibration suppression system on and off lifting continuous (γ = 0°) at 0.7 Ma. (**a**) Response in time domain in pitch plane; (**b**) Response in frequency domain in pitch plane; (**c**) Response in time domain in yaw plane; (**d**) Response in frequency domain in yaw plane.

In order to verify the control effect for the low-frequency and big-amplitude vibration caused by flow separation in pitch plane, conventional tests were first performed with attack angle α continuous ranging from −4° to 10° (roll angle γ = 0°), respectively at 0.6 and 0.7 Ma, the contrast effects with multidimensional vibration suppression system off and on are shown in Figure 11 and Figure 12.

As shown in Figure 11a,c, during the wind tunnel tests, when the aircraft model continuous ranged (roll angle γ = 0°) at 0.6 Ma with multidimensional vibration suppression system off, the aircraft model vibrated violently mainly in pitch plane, and the tests had to stop at 6.98° angle-of-attack. By contrast, with multidimensional vibration suppression system on, the aircraft model did not vibrate obviously within the whole attack angle range from -4° to 10°. The experimental analysis reveals that the useable angle-of-attack reached limiting 10°, increasing by 3.02°. At the same time, the acceleration value reached the maximum of 1.53 g at α = 10°, showing an attenuation of 30.52 g to 4.7% of the previous peak acceleration. As shown in Figure 11b,d, the first modes respectively in pitch plane and yaw plane have been effectively controlled. Similarly, when the aircraft model continuous ranged (roll angle γ = 0°) at 0.7 Ma with multidimensional vibration suppression system on, the aircraft model did not vibrate obviously from −4° to 10°. The experimental analysis reveals that the useable angle-of-attack increased by 2.96° and the acceleration value reached the maximum of 1.29 g at α = 10°, showing an attenuation of 26.21 g to 4.7% of the previous peak acceleration, which is shown in Figure 12a,c. Figure 12b,c shows that the first modes respectively in pitch plane and yaw plane have been effectively controlled. 

**Figure 13:**
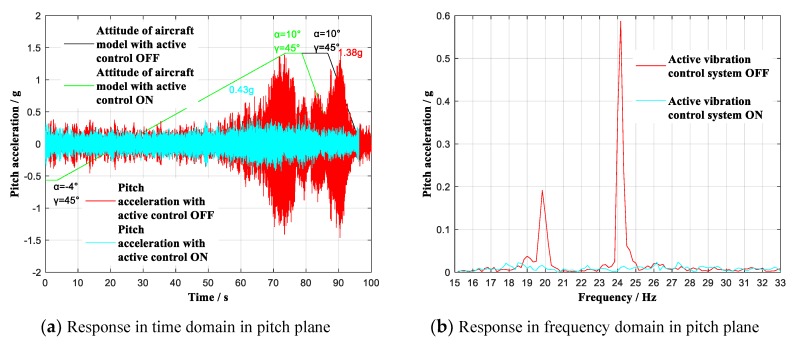

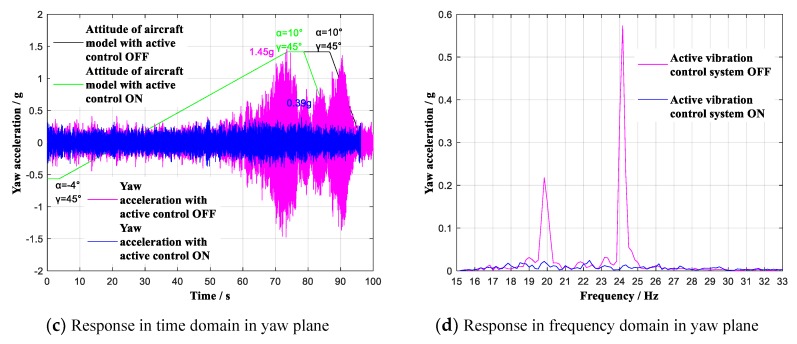
Vibration with multidimensional vibration suppression system off and on lifting continuous (γ = 45°) at 0.6 Ma. (**a**) Response in time domain in pitch plane; (**b**) Response in frequency domain in pitch plane; (**c**) Response in time domain in yaw plane; (**d**) Response in frequency domain in yaw plane.

**Figure 14:**
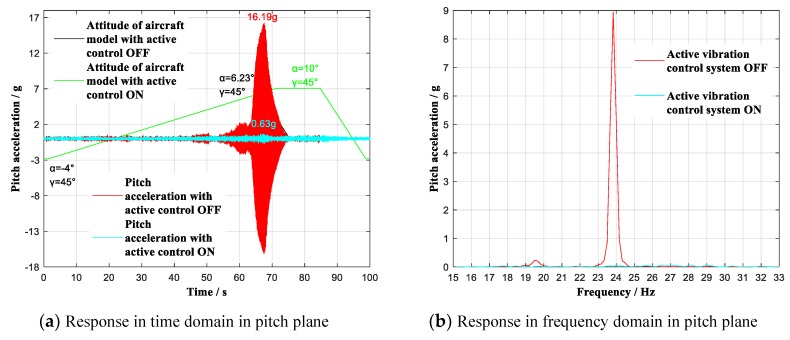

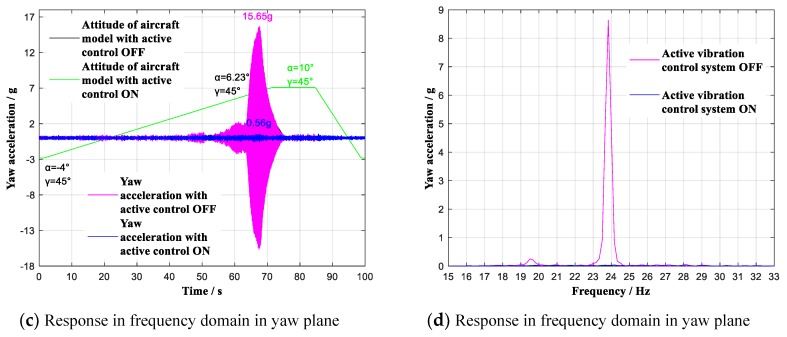
Vibration with multidimensional vibration suppression system off and on lifting continuous (γ = 45°) at 0.7 Ma. (**a**) Response in time domain in pitch plane; (**b**) Response in frequency domain in pitch plane; (**c**) Response in frequency domain in yaw plane; (**d**) Response in frequency domain in yaw plane.

Furthermore, to verify the control effect for adjoint vibration appears in yaw plane, the wind tunnel tests with attack angle α continuous ranging from −4° to 10° (roll angle γ = 45°) were carried out respectively at 0.6 Ma and 0.7 Ma. The contrast effects with multidimensional vibration suppression system off and on is shown in Figure 13 and Figure 14.

As shown in Figure 13a,c and Figure 14a,c, the aircraft rolled to γ = 45°, during the wind tunnel tests, when the aircraft model continuous ranged respectively at 0.6 Ma and 0.7 Ma with multidimensional vibration suppression system off, the aircraft model vibrated equally intensely in pitch plane as well as yaw plane, and the tests had to stop at 6.23° angle-of-attack at 0.7 Ma. By contrast, with multidimensional vibration suppression system on, the aircraft model did not vibrate obviously within the whole attack angle range from −4° to 10°. Specifically, at 0.6 Ma, the experimental analysis reveals that the angle-of-attack smoothly reached limiting 10°. At the same time, the yaw acceleration value reached the maximum of 0.39 g at α = 10°, showing an attenuation of 1.06 g to 26.9% of the previous peak acceleration. At 0.7 Ma, the experimental analysis reveals that the useable angle-of-attack reached limiting 10°, increasing by 3.77°. At the same time, the yaw acceleration value reached the maximum of 0.56 g at α = 6.53°, showing an attenuation of 15.09 g to 3.6% of the previous peak acceleration. As shown in Figure 13b,d, and in Figure 14b,d, the previous order modes in pitch plane and yaw plane were excited, and the first two modes respectively in pitch plane and yaw plane have been effectively controlled.

Based on the verification experiments implemented in wind tunnel above, the verification results are summarized in Table 3. 

In general, for the working conditions (α = −4~10° with γ = 0° or α = −4~10° with γ = 45°) respectively at 0.6 Ma and 0.7 Ma, the spectral analysis of vibrations shows attenuations of more than 26 dB, where the maximum of remainder vibration is 1.53 g less than 5 g. The results have shown that the cantilever sting with multidimensional vibration suppression system can resist the vibration from any direction in wind tunnel tests, and the core measuring equipment can completely stably work inside aircraft model.

## 6. Conclusions

(1) In this paper, a multidimensional vibration suppression method is proposed. With this method, the data quality both in pitch plane and yaw plane is ensured.

(2) On the basis of analysis on the multidimensional active control mechanism, a negative velocity feedback control algorithm combined with a root mean square (RMS) evaluation method is introduced, meanwhile, a weight modification method is performed to determine the sequence number of the stacked piezoelectric actuators and the weight of control voltages in real time. The damping and mass characteristics are strengthened.

(3) A great number of verification experiments were conducted in the lab and a transonic wind tunnel, which indicates that the multidimensional vibration suppression method can resist the vibration from any direction. In the future, we hope to accurately establish the motion equation and further study the active vibration control method with a control model.

## Figures and Tables

**Table 1:** Parameters of stackable piezoelectric actuator.

Parameter	Value	Parameter	Value
Driving Voltage (V)	0–1000	Output Force (N)	5500
Displacement (um)	30	Stiffness (N/um)	190
Diameter (mm)	16	Capacitance (nF)	340
Length (mm)	29 ± 0.5	Resonant Frequency (Hz)	36000

**Table 2:** Verification results in lab.

Impulse Point	Vibration Evaluation Pitch/Yaw	Active Damper ON/OFF	Damping Ratio (Half-Power)	Damping Ratio (Log Decrement)	Spectral Attenuation	Attenuation Time	Natural Frequency
(Pitch Plane) A point	Pitch	OFF	0.003252	0.002124	32DB	33.91 s	25.36 Hz
		ON	0.027258	0.017628		0.69 s	25.01 Hz
	Yaw	OFF	0.003437	0.002209	23DB	9.69 s	25.35 Hz
		ON	0.033048	0.042282		1.44 s	24.93 Hz
(Yaw Plane) B point	Pitch	OFF	0.003575	0.001113	29DB	43.50 s	25.35 Hz
		ON	0.032903	0.011032		1.06 s	24.93 Hz
	Yaw	OFF	0.003627	0.002478	30DB	10.21 s	25.27 Hz
		ON	0.034847	0.021374		0.70 s	24.92 Hz
(Random Plane) C point	Pitch	OFF	0.003144	0.002540	30DB	32.06 s	25.39 Hz
		ON	0.020361	0.012376		1.19 s	25.01 Hz
	Yaw	OFF	0.003457	0.003466	23DB	9.69 s	25.38 Hz
		ON	0.019751	0.008172		1.14 s	25.00 Hz
(Random Plane) D point	Pitch	OFF	0.003159	0.002532	31DB	32.33 s	25.37 Hz
		ON	0.023924	0.015331		1.03 s	25.03 Hz
	Yaw	OFF	0.003746	0.003179	23DB	9.38 s	25.29 Hz
		ON	0.022458	0.013720		1.07 s	25.01 Hz

**Table 3:** Verification results in wind tunnel.

Working Conditions			Vibration Evaluation Pitch/Yaw	Active Damping ON/OFF	Spectral Attenuation	Remainder Acceleration	Improvement
Attack Angle *α*	Roll Angle *γ*	Wind Speed *M*					
−4~10°	0°	0.6 Ma	Pitch	OFF	55 dB	32.05 g	Δα = 3.02°
				ON		1.53 g	
			Yaw	OFF	28 dB	1.75 g	
				ON		0.62 g	
−4~10°	0°	0.7 Ma	Pitch	OFF	50 dB	27.50 g	Δα = 2.96°
				ON		1.29 g	
			Yaw	OFF	26 dB	2.04 g	
				ON		0.77 g	
−4~10°	45°	0.6 Ma	Pitch	OFF	27 dB	1.38 g	Δα = 0°
				ON		0.43 g	
			Yaw	OFF	26 dB	1.45 g	
				ON		0.39 g	
−4~10°	45°	0.7 Ma	Pitch	OFF	44 dB	16.19 g	Δα = 3.77°
				ON		0.63 g	
			Yaw	OFF	48 dB	15.65 g	
				ON		0.56 g	
